# Primary Proximal ACL Repair: A Biomechanical Evaluation of Different Arthroscopic Suture Configurations

**DOI:** 10.3390/jcm12062340

**Published:** 2023-03-17

**Authors:** Steffen B. Rosslenbroich, Andrea Achtnich, Cathrin Brodkorb, Clemens Kösters, Carolin Kreis, Sebastian Metzlaff, Benedikt Schliemann, Wolf Petersen

**Affiliations:** 1 Department of Trauma, Hand and Reconstructive Surgery, University Hospital Muenster, Westfalian Wilhelm’s University, Waldeyerstrasse 1, 48149 Muenster, Germany; 2 Department of Orthopedic Sports Medicine, Klinikum Rechts der Isar, TU Technische Universität Munich, 81675 Munich, Germany; 3 Department of Trauma Surgery and Orthopedics, Maria and Joseph Hospital Greven, 48268 Greven, Germany; 4 Department of Orthopedics and Trauma Surgery, St. Joseph Hospital, 12101 Berlin, Germany; 5 Department of Trauma, Hand and Reconstructive Surgery, Herz-Jesu Hospital, 48165 Münster, Germany; 6 Department of Orthopedics and Trauma Surgery, Martin Luther Hospital, 14193 Berlin, Germany

**Keywords:** ACL repair, Kessler–Bunnell suture, ACL rupture, knotless anchor

## Abstract

**Purpose**: Several suture techniques have been described in the past for direct ACL repair with poor healing capacity and a high re-rupture rate. Therefore, we investigated a refixation technique for acute primary proximal ACL repair. The purpose of this study is to compare the biomechanical properties of different suture configurations using a knotless anchor. **Methods**: In this study, 35 fresh-frozen porcine knees underwent proximal ACL refixation. First, in 10 porcine femora, the biomechanical properties of the knotless anchor, without the ligament attached, were tested. Then, three different suture configurations were evaluated to reattach the remaining ACL. Using a material testing machine, the structural properties were evaluated for cyclic loading followed by loading to failure. **Results**: The ultimate failure load of the knotless anchor was 198, 76 N ± 23, 4 N significantly higher than all of the tested ACL suture configurations. Comparing the different configurations, the modified Kessler–Bunnell suture showed significant superior ultimate failure load, with 81, 2 N ± 15, 6 N compared to the twofold and single sutures (50, 5 N ± 14 N and 37, 5 ± 3, 8 N). In cyclic loading, there was no significant difference noted for the different configurations in terms of stiffness and elongation. **Conclusions**: The results of this in vitro study show that when performing ACL suture using a knotless anchor, a modified Kessler–Bunnell suture provides superior biomechanical properties than a single and a twofold suture. Within this construct, no failure at the bone–anchor interface was seen. **Clinical relevance**: Since primary suture repair techniques of ACL tears have been abandoned because of inconsistent results, ACL reconstruction remains the gold standard of treating ACL tears. However, with the latest improvements in surgical techniques, instrumentation, hardware and imaging, primary ACL suture repair might be a treatment option for a select group of patients. By establishing an arthroscopic technique in which proximal ACL avulsion can be reattached, the original ACL can be preserved by using a knotless anchor and a threefold suture configuration. Nevertheless, this technique provides an inferior ultimate failure load compared to graft techniques, so a careful rehabilitation program must be followed if using this technique in vivo.

## 1. Introduction

The number of injuries of the anterior cruciate ligament (ACL) requiring surgery in the United States is reported to be between 80,000 and 200,000 [1,2] per year and steadily increasing. This frequent injury has been well-known since ancient times, first described by Galen over 2000 years ago [3]. Knowledge about the function of the ACL and the possible consequences in case of a rupture were obtained much later. The first ACL surgery was reported by M. Robson [4] in 1895, treating a miner with ACL and posterior cruciate ligament (PCL) rupture using primary open suture. Initially, good results were reported but could not be maintained over time. As the necessity of ACL reconstruction was questioned in the following decades and long-term results showed deterioration, orthopedic surgeons abandoned the technique of suturing the ACL and turned to reconstruction. Grekow is considered to be the first surgeon to use autologous tissue for ACL rupture reconstruction in 1914 [3]. Since then, autologous or allogenic tissue has become the gold standard [5]. In the 1970s, Feagin et al. [6] tried to restore the repair of the torn ACL but demonstrated unsatisfying long-term results. At that time, several authors [7,8,9] reported modified ACL repair techniques with inconsistent results and failure rates. Analyzing these historical results of primary ACL repair, it must be mentioned that a clear description of indication, diagnosis, concomitant injuries and type of ACL pattern is lacking and might be responsible for heterogeneous results. Nevertheless, current improvements in diagnostic, imaging and operation techniques might reduce the risk of failure by careful patient selection. We consider patient and injury selection to be essential for favorable outcomes in ACL repair, since the compliance of the patient and healing potential of the torn ligament and rupture pattern have a great influence on the failure rate. One of the most important aspects to consider is the favorable vascularity in the proximal third of the acl [10,11]. Keeping this in mind, we believe that primary repair is a reliable option for treating a select group of patients with ACL injuries. Successful ACL repair might reduce the number of patients needing ACL reconstruction [12].

Another relevant aspect is that the proprioceptive nerve fibers of the torn ACL are usually not reproduced by the inserted graft [13].

Regarding the prognosis of the ACL tear type, the histological study of Nguyen et al. [14] is relevant. The authors investigated the reattachment of the tibial remnant of the torn ACL to the posterior cruciate ligament (PCL) and compared the intrinsic healing response of the ACL with the histological characteristics of the medial collateral ligament (MCL). They presumed that the proximal ACL tissue has a similar healing response to the MCL. Based on these findings, we developed our knotless suture anchor ACL repair technique to reattach proximal avulsion ACL tears. Recently, DiFelice et al. [10] and Weninger et al. [15] described a similar technique for proximal ACL repair using two anchors for a double-bundle repair technique.

The purpose of this investigation is to biomechanically evaluate different arthroscopic suture-repair configurations for ACL ruptures using a knotless anchor to ensure the optimal reattachment of the proximal avulsion of the ACL tear.

Since new fixation devices, especially in knee and shoulder surgeries, have since been developed, and new knowledge of knee biomechanics and anatomy has since been discovered, this might provide a reasonable basis for re-evaluating the approach of M. Robson in 1895.

Another aspect that supports the need for such a technique is the fact that meniscal tears are frequently associated with a ruptured ACL. In particular, the dislocated bucket-handle meniscal tear is an urgent cause for surgery. If, however, the knee seems to be in an inflammatory phase after injury with swelling and without full range of motion, there is a high risk of postoperative arthrofibrosis if ACL reconstruction using an autologous graft is performed [16]. It is widely accepted that, in this acute phase, the meniscal repair is performed at an early stage and the ACL reconstruction at a later stage if necessary. The disadvantages of this approach are the two necessary surgeries instead of one and that, due to the persisting instability after meniscal repair, the outcome might also become worse than it would with simultaneous ACL reconstruction providing a stable situation. Although Kwok et al. [17] slightly relativized this statement, it is still common practice to wait for full range of motion and for minimal swelling. To provide stability in a knee receiving a meniscal refixation that is also in an inflammatory phase after injury, we aim to address an ACL rupture without the need for a biological graft and excessive drilling to minimize the risk of postoperative arthrofibrosis.

## 2. Materials and Methods

In this study, 35 fresh-frozen porcine knees were used. The mean age of the animals was 28 weeks ± 2 weeks. The knees were stored at −20 °C until 5 h prior to testing. Before testing, the specimens were removed from the freezer, thawed and moistened. The adherent soft tissue was removed with special care to ensure the integrity of the ACL. After cutting the ACL at the femoral insertion, the tibia was separated from the femur, imitating a Sherman Type I femoral ACL avulsion [18]. The tests were performed at room temperature and the ACL was kept moist with saline solution during mounting and testing to prevent desiccation.

### 2.1. Test Set-Up and Loading Protocol

**Figure 1:**
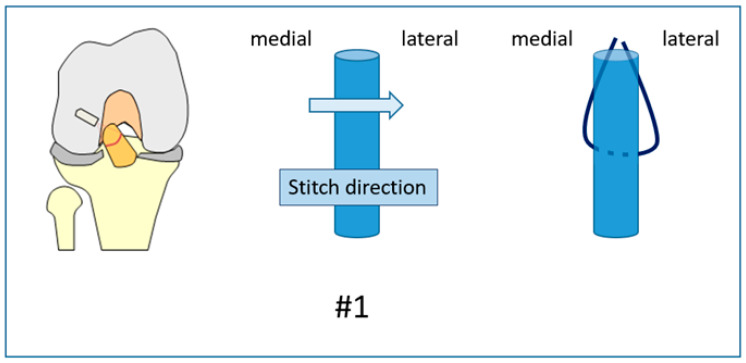
Suture #1 was a simple stitch passing the suture from the medial aspect of the ACL to the lateral.

**Figure 2:**
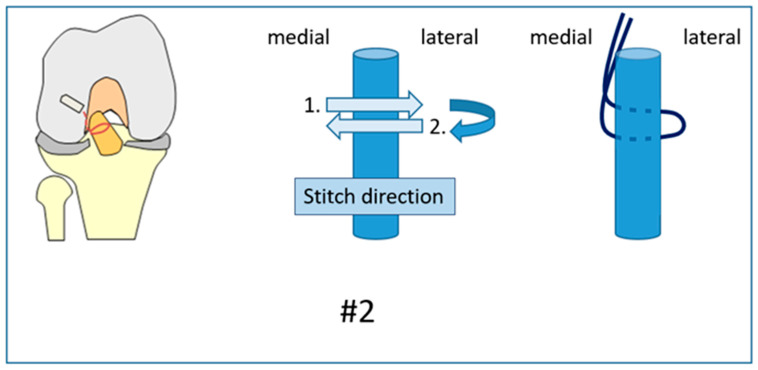
Suture #2 was the same stitch but returning at an angle of 180°, passing twice through the ACL.

**Figure 3:**
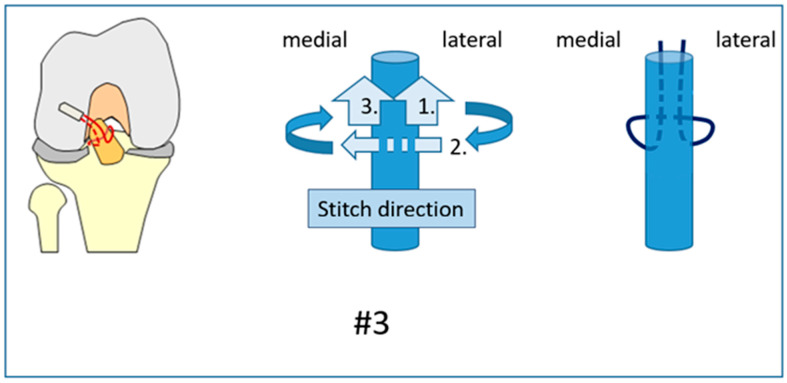
Suture #3 was performed as a modified Kirchmayr [21] suture.

Three different suture configurations were tested (Figure 1, Figure 2 and Figure 3). In suture group I, 10 specimens were tested, 13 specimens were tested in group II and 12 specimens were tested in group III. The tibia with adherent ACL was mounted to the base of the testing frame. Tensile testing was performed using a ZWICK uniaxial material testing machine (Zwick/Roell Z005; Zwick, Ulm, Germany). The tibia with adherent ACL was fixed in the frame in an angle of 30° to imitate the conditions of a clinical Lachman test. A customized suture clamp was attached to the cross-head to hold the proximal end of the suture.

For the evaluation of the biomechanical properties of the knotless anchor (Arthrex, Naples, FL, USA), tensile testing was performed using ten porcine femora. For this, the same test set was used, using a femur instead of the tibia with no adherent ACL. The suture of the anchor was fixed to the prior mentioned suture clamp.

After preconditioning the construct with 10 cycles and a load of 5–10 N at a cross-head speed of 200 mm/min, each tibia/ACL suture (femur/anchor) construct was subjected to 1000 cycles of a load between 5 and 10 N at a cross-head speed of 200 mm/min, and subsequently the construct was loaded to failure. The displacement rate of 200 mm/min is thought to be reflective of in vivo loading forces and has been used previously [19,20]. The number of cyclic loads is also in accordance with previously published studies [19,20]. Subsequently, ultimate load-to-failure testing was performed using the same cross-head speed. Load elongation was recorded continuously. To determine the structural properties of each suture configuration and the construct`s stiffness, the linear region of the load elongation curve, ultimate failure load and the mode of failure were documented.

Statistical analysis was performed with SPSS Version 11.0 using the Kruskal–Wallis and the Mann–Whitney U tests.

The evaluation and presentation of the data, as well as the determination of mean values and standard deviations, was carried out using the “Excel” program (Microsoft, Redmond, DC, USA).

The statistical analysis of the results obtained as part of the study was carried out using the statistics program SPSS 12 for Windows (SPSS, Munich, Germany). The Kruskal–Wallis test was used.

The level of significance was defined as *p* ≤ 0.05 and, if a significant difference was detected within the samples, it was determined using the Wilcoxon–Mann–Whitney test in multiple direct comparisons of the samples.

### 2.2. Surgical Technique

For the tensile testing of the knotless anchor (PushLock Arthrex, Naples, FL, USA), a 3.5 mm drilling guide was placed at the anatomic insertion of the ACL. Beforehand, the two ends of the suture (Fiberwire^®^, 2/5 metric, 38“ Arthrex, Naples, FL, USA) were passed through the suture clamp, leaving a loop of 3 cm length. Thereafter, the anchor was introduced using the insertion device (Arthrex, Naples, FL, USA) into the drill hole until the marked spot on the introducer was reached. Then, with the gentle use of a hammer, the screw was introduced to secure the loop in the anchor.

## 3. Results

### 3.1. Elongation after 1000 Cycles

**Figure 4:**
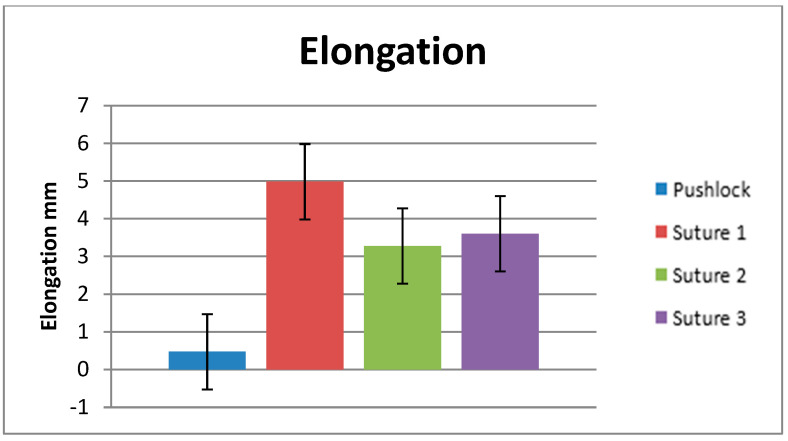
Elongation.

Every anchor construct survived the cyclic loading protocol of 1000 cycles between 5 and 20 N, showing a mean elongation of 0.47 mm (±0.13 mm) (Figure 4: Elongation). Of the single sutures, 70% failed during cyclic loading at a mean of 197 cycles (±294). For the remaining 30%, the mean elongation after 1000 cycles was 4.9 mm (±1.5 mm). A total of 7.8% of the specimens of Suture #2 failed during cyclic loading. The surviving specimens showed a mean elongation of 3.6 mm (±0.6 mm). All sutures in group #3 survived the cyclic loading, showing a mean elongation of 3.3 mm (±0.99 mm). No significant differences comparing the different suture configurations in terms of elongation were found (*p* = 0.075).

### 3.2. Structural Properties

**Figure 5:**
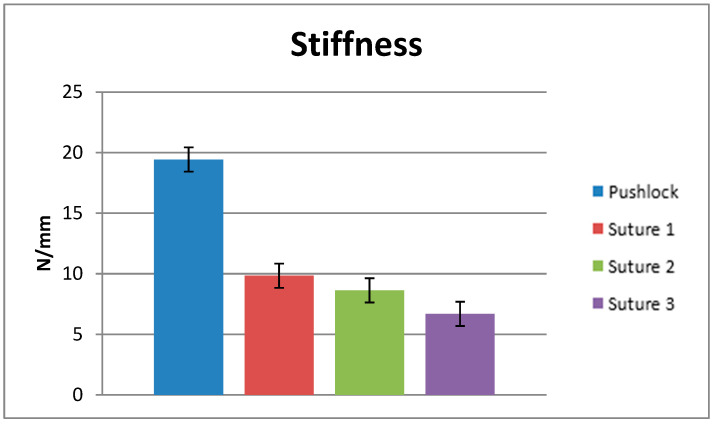
Stiffness.

Looking at the stiffness testing in the anchor group, a mean of 198.76 N (±23.44) was achieved. Comparing the anchor in terms of stiffness to the sutures used, significant differences were found for every group (*p* < 0.05) (Figure 5: Stiffness). Suture #1 showed a stiffness of 9.8 N/mm (±2.6 N/mm); Suture #2 a mean of 8.6 N/mm (±3.4 N/mm); and Suture #3 a mean of 6.6 N/mm (±2.1 N/mm). Between the sutures used, there was no significant difference found (*p* > 0.05).

**Figure 6:**
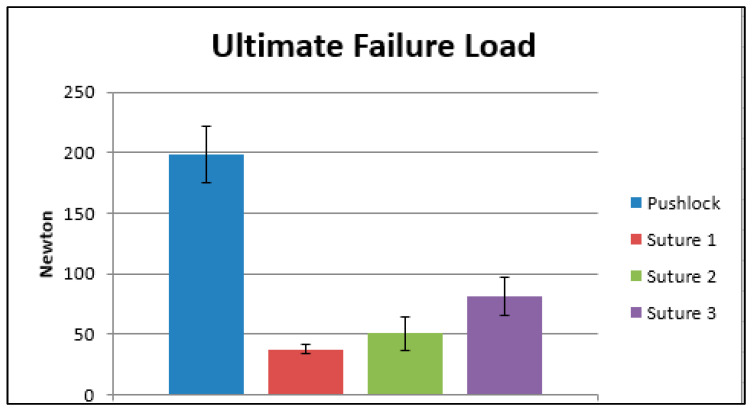
Ultimate Failure Load.

The ultimate failure load of the anchor (Figure 6: Ultimate Failure Load) showed a mean of 198, 7 N (±23, 4 N). Significant differences (*p* < 0.05) were found in every comparison of the sutures. Suture #1 showed a mean ultimate failure load of 37.5 N (±3.8 N); #2 a mean of 50.5 N (±14.2 N); and Suture #3 revealed an ultimate failure load of 81, 2 N (±15, 6 N).

### 3.3. Failure Mode

The anchor testing failed at the ultimate load due to the suture being pulled out of the anchor. All suture repairs showed a failure mode by the suture cutting through the ACL. The suture (Fiberwire^®^, 2/5 metric, 38“ Arthrex, Naples, FL, USA) showed no signs of failure.

## 4. Discussion

The aim of this study was to evaluate the biomechanical properties of three suture configurations using a knotless anchor refixation for the proximal avulsion of the ACL. Comparing the in vitro results within this study, we see evidence that the use of a modified Kirchmayr [21] technique is preferable and shows the least risk of failure within the different suture configurations. Using a one-stitch suture, only 7.2% of the specimens survived the cyclic loading protocol. The suture material and the anchor fixation were able to withstand all the forces applied in this study. The weakest link in this construct appeared to be the suture cutting through the ACL. In terms of ultimate failure load, significant advantages for the modified Kirchmayr [21] technique were found compared to the two other configurations.

Compared with the conventional ACL reconstruction technique as operative alternative options, there are some advantages and disadvantages to discuss. Despite excellent clinical results, failure rates of up to 7.7% are reported [22,23,24] and up to 25% in adolescents [12]. Looking further at skeletally immature patients, the treatment path to choose in patients with open physes is still unclear. Reports exist about the risk of limb length deformity using transphyseal grafts [25,26]; therefore, a repair technique not involving the open physes would be favorable [27]. Additionally, long-term results still show a high incidence of osteoarthritis despite ACL reconstruction [28,29,30]. Murray et al. [31] recently reported in their investigation of a porcine model that primary ACL repair reduces the magnitude of osteoarthritic changes when compared with other surgical stabilization procedures.

Even though the current techniques of ACL reconstruction using autologous grafts such as BPTB or hamstrings or allografts show satisfying results, certain conditions have a large influence on the timing and outcome of these procedures. Other aspects which have an influence on the clinical outcome after ACL reconstruction are harvest-site morbidity and proprioceptive deficits [32].

To rule out harvest-site morbidity and to preserve the proprioceptive tissue in the original ACL, a technique of repairing the torn ACL would be favorable. Furthermore, this repair would enable an orthopedic surgeon to provide stability in knees receiving a meniscal refixation and in an inflammatory phase after injury, possibly reducing the risk of postoperative arthrofibrosis.

Since the attempts of Feagin et al. in the 1970s [33], findings in the understanding of the torn ACL in terms of rupture patterns, biomechanical aspects and knowledge concerning the healing process enable us to re-evaluate the approach of primary repair of the torn ACL. Furthermore, the development of new fixation devices and techniques supports this approach. Reviewing the approach of Feagin et al. [6], Difelice et al. [10] suggest that with modern imaging options and modern arthroscopic techniques, a more thorough patient selection receiving primary ACL repair might result in a favorable outcome. In their study, DiFelice et al. [10] proposed a suture anchor repair for ACL rupture, retrospectively looking at 11 patients. The authors emphasize the importance of the rupture pattern, taking the classification by Sherman et al. [18] and the tissue quality of the torn ACL into account. The authors used a 3–4 stitch locking Bunnell-type suture for the antero-medial bundle and the postero-lateral bundle, fixing each bundle with a 4.75 mm BioComposite SwiveLock suture anchor (Arthrex, Naples, FL). By limiting the indication for primary anchor repair of the ACL to a Sherman Type I femoral avulsion with good tissue quality, the authors were able to show a good clinical and subjective outcome in 10 of 11 patients at a mean follow up period of 3.5 years with a mean Lysholm Score of 93.2 and a mean subjective IKDC Score of 86.4.

Taking the aspect of bone marrow stimulation into account to promote ACL healing as described by Steadman et al. [34,35], Gobbi et al. [36] treated 26 patients with an incomplete ACL rupture using a Duncan loop [37] to adapt the rupture site to the residual ACL part with additional microfracturing at the femoral footprint. Using this technique, the authors showed a significant reduction in the mean rollimeter side-to-side difference from 3.5 mm to 1.3 mm postoperatively at a mean follow up of 25.3 months, concluding that young athletes with acute incomplete ACL lesions treated with primary repair and bone marrow stimulation show satisfactory knee stability.

Systematically reviewing the literature concerning the topic of primary ACL repair, Taylor et al. [38] tried to determine whether there is still a role for the primary repair of the ACL among a highly selected group of patients. They identified eight clinical human studies that focused on primary repair of the ACL. Within the clinical studies, four studies [39,40,41,42], including the evaluation [41] of Feagin et al. [6] from the 1970s, reported unacceptable long-term results when performing an arthrotomy and a transosseous suture configuration with prolonged cast immobilization for primary ACL repair. In terms of studies dealing with an arthroscopic repair technique, the authors identified four studies, of which Toy et al. [43] and Ahn et al. [44] described one case each of a tibial-sided soft-tissue avulsion of the ACL with satisfactory results. Arbes et al. [45] reported three patients with primary repair, of which two ultimately underwent ACL reconstruction and the remaining patient had a poor clinical outcome. Gaulrapp et al. [46] evaluated skeletally immature patients who were treated for ACL rupture using primary repair or reconstruction. Forty-four adolescents were available for follow-up, five of whom had a primary ACL repair performed. The authors conclude, due to the inferior clinical outcome of the primary repair compared to the reconstruction of the ACL, that reconstruction in adolescents is superior to primary repair. Achtnich et al. showed, in a comparative study with a mean follow-up of 28 (24–31) months, no significant differences when comparing 20 patients who underwent ACL repair and 20 patients who underwent ACL reconstruction. No significant differences were detected for KT-1000, Lachman, Pivot-shift and IKDC Score. Although there was a failure rate of 15% in the repair group, this was not statistically significant (*p* = 0.231) [47].

To the best of our knowledge, this is the first biomechanical investigation of different suture configurations in primary knotless anchor repair. These results may be important for the development of arthroscopic suture techniques for primary ACL repair. We believe that the approach by Difelice et al. [10] of taking the rupture pattern and the tissue quality into consideration is essential for favorable clinical results. Furthermore, we agree with Gobbi et al. [36] that microfracture at the femoral footprint seems to have benefits for healing.

### Limitations

Several limitations apply to this study since we applied a perpendicular force to the knee joint in the Lachman position on the ACL, imitating a worst-case scenario; this might not fully be representative of the actual force within the knee causing an ACL suture to fail. The biomechanical values that were shown in this study may not be taken as an approximation of the in vivo circumstances. However, we emphasize the comparative character of this study since the same material and set-up were used for all the different suture configurations.

Looking at the test set-up, we tried to rule out further variables by cutting the ACL and providing a comparable injury pattern. Cutting the ACL does not resemble the ACL rupture pattern, which is mostly an avulsion. By doing so, we focused on the comparison of the suture techniques and created equal conditions.

Furthermore, since this is an in vitro study, biological aspects that influence the healing in vivo, e.g., the blood supply at the femoral footprint of the ACL that might be compromised due to the grasping suture configuration, were not taken into account. This study provides data for the primary stability of ACL suture repair that we consider as highly important for the healing of the ligament. Due to its in vivo set-up, this study cannot provide a statement relating to healing in the medium or long term.

## 5. Conclusions

To answer the question posed by Taylor et al. [38] of whether or not there is still a role for the primary repair of the ACL, further clinical studies with higher numbers of patients must be performed in order to determine whether to promote or to abandon primary ACL repair.

To the best of our knowledge, our study is the first that examined a biomechanical point of view of the suture configuration for use in the primary repair of proximal ACL avulsions. As further information is needed to promote primary ACL repair, the biomechanical data of the modified Kirchmayr [21] suture with the use of a knotless anchor shown in this study can serve as the basis for upcoming clinical studies in our departments.
